# High-Frequency Sheet Conductance of Nanolayered WS_2_ Crystals for Two-Dimensional Nanodevices

**DOI:** 10.1021/acsanm.2c03517

**Published:** 2022-10-13

**Authors:** Stan E.T. ter Huurne, Adonai Rodrigues Da Cruz, Niels van Hoof, Rasmus H. Godiksen, Sara A. Elrafei, Alberto G. Curto, Michael E. Flatté, Jaime Gómez Rivas

**Affiliations:** † Department of Applied Physics and Eindhoven Hendrik Casimir Institute, Eindhoven University of Technology, P.O. Box 513, Eindhoven5600 MB, The Netherlands; ‡ Department of Physics and Astronomy, University of Iowa, Iowa City, Iowa52242, United States

**Keywords:** terahertz near-field spectroscopy, terahertz conductivity, electron−phonon coupling, tungsten disulfide, transition metal dichalcogenide, density functional theory

## Abstract

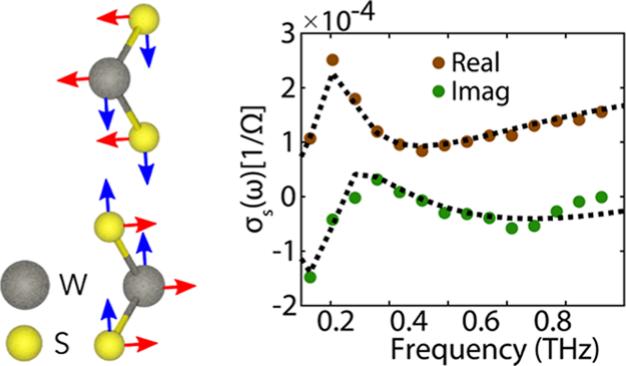

Time-resolved terahertz (THz) spectroscopy is a powerful technique for the determination of charge transport properties in photoexcited semiconductors. However, the relatively long wavelengths of THz radiation and the diffraction limit imposed by optical imaging systems reduce the applicability of THz spectroscopy to large samples with dimensions in the millimeter to centimeter range. Exploiting THz near-field spectroscopy, we present the first time-resolved THz measurements on a single exfoliated 2D nanolayered crystal of a transition metal dichalcogenide (WS_2_). The high spatial resolution of THz near-field spectroscopy enables mapping of the sheet conductance for an increasing number of atomic layers. The single-crystalline structure of the nanolayered crystal allows for the direct observation of low-energy phonon modes, which are present in all thicknesses, coupling with free carriers. Density functional theory calculations show that the phonon mode corresponds to the breathing mode between atomic layers in the weakly bonded van der Waals layers, which can be strongly influenced by substrate-induced strain. The non-invasive and high-resolution mapping technique of carrier dynamics in nanolayered crystals by time-resolved THz time domain spectroscopy enables possibilities for the investigation of the relation between phonons and charge transport in nanoscale semiconductors for applications in two-dimensional nanodevices.

## Introduction

Transition metal dichalcogenides (TMDs) are a class of materials consisting of van der Waals stacked 2D layers with the chemical formula MX_2_, with M a transition metal atom (Mo or W) and X a chalcogen atom (S, Se, or Te). The electronic band structure of TMDs changes from indirect to direct semiconductor as the dimensionality is reduced from the bulk to the monolayer,^1^ which has led to intensive research over the past years.^2^ For some monolayer and few-layer TMDs, the difference in exciton populations in the K- and K’-points of the hexagonal Brillouin zone leads to a new degree of freedom in charge transport and gives rise to the so-called valleytronics,^3^ which allows the control of electron spin by the handedness of the excitation light.^4^ The quantum confinement of charges in low-dimensional TMDs leads to a very strong exciton binding energy as well, which translates into an extraordinarily high absorptance.^5^ These properties make low-dimensional TMDs potentially interesting candidates for various applications in nanoelectronics and optoelectronics, such as transistors, optical switches, photodetectors, and light emission.^5−9^ So far, the highest quality low-dimensional TMDs are obtained by the mechanical exfoliation of nanolayered crystals from the bulk material. The way these nanolayered crystals are transferred to other substrates and how the samples are further prepared have a tremendous impact on the electrical and optical properties of the nanolayered crystals.^10^ Therefore, the detailed non-invasive characterization of TMDs to retrieve quantities such as carrier mobility, density, or lifetime is of utmost importance for their applicability in two-dimensional nanodevices. Also, the investigation of low-energy phonon modes is of relevance since electron–phonon coupling in crystals could influence the conductance in TMDs, as has been proposed in van der Waals bound organic semiconductors.^11^

A powerful technique for the investigation of charge transport in semiconductors is time-resolved terahertz (THz) spectroscopy (TRTS). This time domain technique is capable of directly probing the frequency-dependent carrier dynamics of TMD layers non-invasively and contact free.^12,13^ With this technique, the high-frequency conductivity of the material is probed instead of the DC conductivity that is typically retrieved using electrical contacts. However, the long wavelengths of THz radiation limit the applicability of TRTS to large samples that are grown by chemical vapor deposition, molecular beam epitaxy, atomic layer deposition, or spray-coated flakes where microscopic defects are averaged.^14−16^ These grown samples have also a lower quality than exfoliated nanolayered crystals, which implies that properties retrieved from the former cannot be extrapolated to the latter.

Here, we introduce time-resolved THz near-field spectroscopy to retrieve the THz sheet conductance with sub-diffraction spatial resolution. This technique allows us to perform the first THz measurements of the frequency-dependent pump-induced sheet conductance of a single exfoliated nanolayered crystal of WS_2_ with varying thickness. The sheet conductance drops for all measured thicknesses and shows a specific frequency dependence. Using density functional theory (DFT), we have identified that this frequency dependence is caused by the electron–phonon coupling between charge carriers and the fundamental breathing mode in WS_2_.^17^ The single-crystal structure of the exfoliated nanolayered crystal enabled us to characterize the breathing phonon mode at 0.2 THz. Furthermore, the calculations show the strong effect of strain in the crystal structure on the phonon mode, which can be associated with a substrate on which the nanolayered crystal is deposited. These results represent the first demonstration of contact-free and high-resolution THz measurements of carrier properties in exfoliated nanolayered TMDs. The coupling of low-energy phonon modes to free charges is shown, and how this coupling influences the conductance of nanolayered single-crystalline WS_2_, affecting their applicability for two-dimensional nanodevices.

## Results

### Time-Resolved THz Near-Field Microscopy

**Figure 1:**
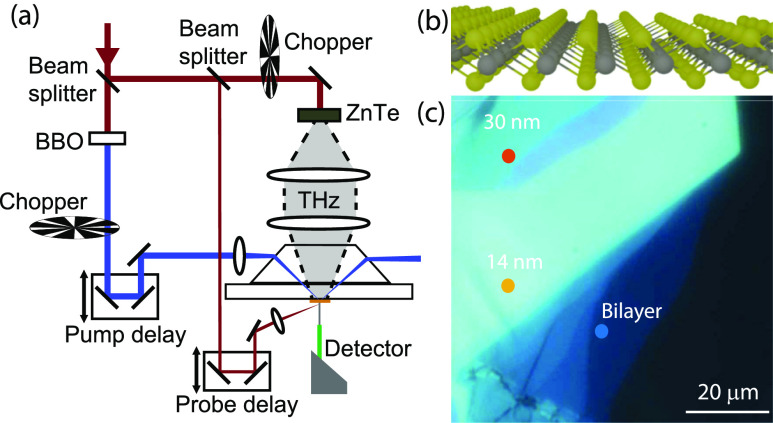
(a) Schematic representation of the time-resolved THz near-field microscope. The exfoliated nanolayered crystal is on a thin layer of PDMS on a quartz substrate, and it is optically excited from the back through the Dove prism. The microprobe detector is positioned at a distance less than 1 μm away from the nanolayered crystal to measure the transmitted THz amplitude with sub-diffraction spatial resolution upon photoexcitation of the nanolayered crystal. The angle of optical pump excitation is larger than the critical angle for total internal reflection, such that no pump intensity is transmitted and reaches the THz microprobe detector. (b) Crystal structure of a single atomic layer of WS_2_. (c) Reflection optical microscopy image of the exfoliated nanolayered crystal, the dots indicate the measured positions corresponding to a bilayer and two thicker regions.

The measurements are performed with a sub-diffraction time-resolved near-field THz microscope schematically illustrated in Figure 1a (see Methods section for a full description).^18^ The setup measures the local electric field amplitude with a THz near-field photoconductive antenna (microprobe detector), which has a high spatial resolution of ≃15 μm, equal to λ/40 at a wavelength of maximum amplitude. To inject charge carriers, the sample is optically excited with femtosecond optical pulses from an amplified laser system at a central wavelength of 400 nm through a Dove prism at a large angle, ensuring total internal reflection at the sample–air interface. This configuration prevents the optical pump to reach the microprobe detector. The quartz substrate is brought in optical contact with the Dove prism of the THz spectrometer using a refractive index matching liquid at optical frequencies to allow for optical excitation from the back.

### Nanolayered WS_2_ Crystal

The sample used for the experiments is a single exfoliated nanolayered crystal of tungsten disulfide, WS_2_, whose crystal structure is shown in Figure 1b. To measure this nanolayered crystal, we first place a polydimethylsiloxane (PDMS) film (Gel Pak, PF-80-X4, 6.5 mil) on a crystalline quartz substrate. Then, nanolayered WS_2_ crystals are deposited onto the PDMS film by mechanical exfoliation from synthetic 2H-WS_2_ crystals (HQ Graphene) using the Nitto SPV224 tape.

An optical microscopy image of the exfoliated nanolayered WS_2_ crystal is shown in Figure 1c. The colored dots indicate the positions where measurements of the photoinduced sheet conductance were performed, as discussed in the next sections. The region at the edge of the nanolayered crystal contains a large bilayer region (1.4 nm thick, blue dot). Next to the bilayer, there is a thicker region of 14 nm (yellow dot). The thickest region is at the top left and is approximately 30 nm thick (red dot). The thickness of the bilayer region is determined by the peak position of the indirect band gap emission (Figure S1), which is very sensitive to thickness in the region of one to five atomic layers. For a bilayer, the indirect emission peak is located around 705 nm, whereas for increasing thickness, it shifts to longer wavelengths.^3,19^ The thicker regions have been measured using atomic force microscopy (Figure S2).

### Photoinduced Transmittance and Sheet Conductance of Nanolayered WS_2_ Crystals

**Figure 2:**
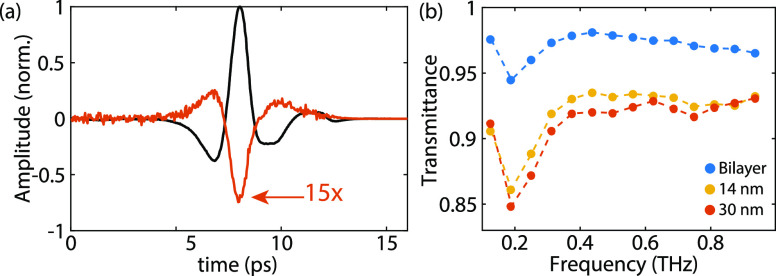
(a) Normalized electric field amplitude of a THz transient (black) and the photoinduced change of the transient upon photoexcitation (red, amplified 15 times). (b) Photoinduced change in transmittance as a function of frequency for the three measured thicknesses.

A measurement of the THz transmission in the time domain, that is, a THz transient, is shown in Figure 2a for the 30 nm-thick layer (black curve). A digital truncation is introduced in the transient after 12 ps to suppress etalons originating from internal reflections in the microprobe detector and to simplify data processing. Upon photoexcitation, the transmitted THz radiation will be reduced due to additional free carrier absorption in the excited nanolayered crystal. By comparing the transmitted radiation with and without photoexcitation, the properties of the photoinduced carriers can be investigated. The photoinduced change of the THz pulse, 20 ps after photoexcitation (incident pump fluence 20 μJ/cm^2^), is presented in Figure 2a for the 30 nm-thick area (red curve, multiplied 15 times for clarity). The photoinduced change in amplitude is similar in shape to the transmitted THz pulse but of opposite sign, meaning that for the entire pulse, the response due to the presence of carriers is similar.

The frequency-dependent complex transmission coefficient can be calculated by Fourier transforming the THz transient signals and normalizing the photoexcited to the non-excited measurements. The transmittance (squared modulus of the complex transmission coefficient) is plotted in Figure 2b as a function of frequency for the three WS_2_ layer thicknesses. The measurements present a clear thickness dependence on the transmittance, as in a thicker layer, more photoexciting light is absorbed, generating more free carriers that attenuate the THz radiation. A sharp increase in transmittance between 0.2 and 0.4 THz is visible, after which the response becomes less frequency dependent.

To quantify the photoinduced change on the nanolayered WS_2_ crystal, the measurements need to be converted to a material parameter. Since the photoinduced change in transmittance is caused by the increase of free carriers in the material, it contributes directly to the photoinduced conductivity. The photoexcited layer can be approximated by a uniform thin film with a specified photoinduced sheet conductance because the photoexcited layer is thin compared to the THz wavelength (*h*/λ ≪ 1). The photoinduced sheet conductance can be calculated from the complex transmission coefficient using the thin-film approximation^20,21^
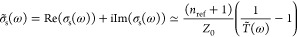
1Here, *n*_ref_ is the refractive index of the WS_2_ layer without photoexcitation (2.28),^22^*Z*_0_ is the impedance of free space (377 Ω), and  is the photoinduced change in the complex transmission coefficient.

**Figure 3:**
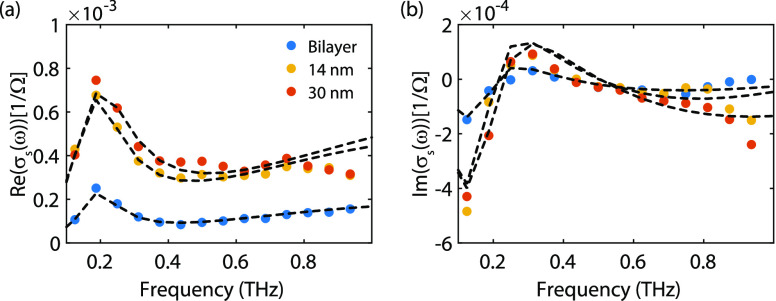
Sheet conductance fitted with the Drude–Smith model with a phonon contribution for the three measured thicknesses for the real (a) and imaginary component (b).

The real and imaginary components of the sheet conductance are depicted in Figure 3a,b for the bilayer (blue circles), the 14 nm (yellow circles), and 30 nm (red circles) thick regions of the nanolayered crystal. Upon photoexcitation, free carrier absorption is the dominant contribution to the reduced transmittance. Since an increased free carrier absorption leads to a larger sheet conductance, there is an inverse relation between the sheet conductance and the transmittance.

The Drude–Smith model with a Lorentzian phonon contribution is used to fit this photoinduced sheet conductance and retrieve the free carrier properties:^23,24^
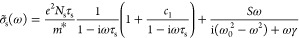
2where *N*_s_ is the photoinduced sheet carrier density, τ_s_ is the carrier scattering time, m* is the carrier effective mass (0.4 *m*_e_, with *m*_e_ the electron mass),^25^ and *c*_1_ is the Smith contribution describing the degree of backscattering at each scattering event (*c*_1_ = −1 corresponds to pure backscattering and *c*_1_ = 0 to random scattering events, i.e., Drude).^26^ The second term in eq 2 is a Lorentzian that characterizes a phonon contribution to the sheet conductance. In this term, *S* is the oscillator strength, ω_0_ is the phonon frequency, and γ is the lifetime broadening parameter.^23,26^

The fits, plotted in Figure 3 with dashed curves, are in excellent agreement with the data for both the real and imaginary components. The fitted parameters given in Table 1 show a larger photoinduced sheet carrier density with the number of layers due to an increased absorption. The carrier scattering times have similar values of 100 fs, which are in agreement with values reported in bulk TMDs.^23^ The backscattering contribution is ≃-1 and originates from the van der Waals stacking of WS_2_ layers. This stacking suppresses free carrier scattering between atomic layers. Furthermore, due to mechanical exfoliation, there are more defects on the surface (where most light is absorbed), leading to an even larger backscattering contribution.^27^ The phonon resonance has a frequency around 0.2 THz, which corresponds to a breathing phonon resonance.^17^ This breathing phonon mode, which we confirm with DFT in the next section, is responsible for the THz sheet conductance peak that we experimentally retrieve with THz near-field microscopy.

**Table 1:** Values of the Parameters from the Sheet Conductance fits. The Uncertainty Is Given by the 95% Confidence Interval of the Fit

thickness	*N*_s_(cm^–2^)	*τ*_*s*_(fs)	*c*_1_	ω_0_/2π(THz)	S (s/Ω)	γ/2π (THz)
bilayer	(0.48 ± 0.16)10^13^	110 ± 23	–1 ± 0.12	0.19 ± 0.01	(15 ± 6)10^4^	0.16 ± 0.05
14 nm	(1.3 ± 0.5)10^13^	107 ± 26	–1 ± 0.17	0.18 ± 0.01	(5.5 ± 2.5)10^4^	0.19 ± 0.06
30 nm	(2.1 ± 0.9)10^13^	85 ± 35	–0.98 ± 0.12	0.20 ± 0.01	(3.0 ± 1.2)10^4^	0.22 ± 0.06

### DFT Calculations of Interlayer Phonons

**Figure 4:**
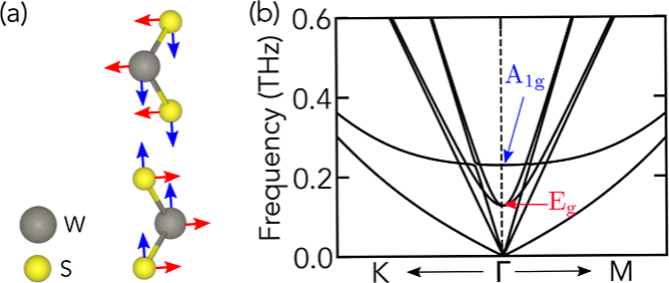
(a) Conventional unit cell of bilayer WS_2_. Blue arrows indicate the relative movement between layers for the breathing mode (A_1g_) and red arrows for the shear mode (E_g_). (b) Phonon dispersion around the Γ point highlighting low-frequency breathing and shear modes for a WS_2_ bilayer.

The phonon modes of WS_2_ bilayers were obtained from first-principles self-consistent calculations with the Vienna Ab Initio Simulation Package (VASP),^28−30^ as described in the Methods section. A conventional unit cell for the 2H-polytype bilayer with two W and four S atoms (Figure 4a) was used in all the calculations. The unit cell of the WS_2_ bilayer belongs to the D_3d_ point group.^31,32^ The six atoms of the unit cell generate 18 vibrational modes that are classified according to the irreducible representations of its point group at the Γ point, Γ^*vib*^ = 3(A_1g_ + A_2u_ + E_g_ + E_u_), where one A_2u_ and one E_u_ are acoustic modes, the other A_2u_ and E_u_ are IR active, and the A_1g_ and E_g_ are Raman active associated with the out-of-plane breathing mode (blue arrows in Figure 4a) and the in-plane shear mode (red arrows in Figure 4a), respectively. As a consequence of the relatively weak interlayer van der Waals interactions, each layer moves almost rigidly as a whole unit, giving rise to low-frequency modes corresponding to the vibrations between layers dominated by interlayer restoring forces.^33^ The phonon dispersion around the Γ point for a bilayer WS_2_ under vacuum is shown in Figure 4b, the A_1g_ and E_g_ phonon modes occur around 0.225 THz and 0.124 THz, respectively.

**Figure 5:**
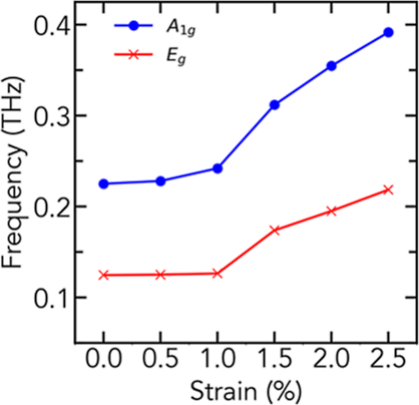
Energy of the A_1g_ and E_g_ phonon modes as a function of biaxial compressive strain at the Γ point.

The calculated breathing phonon frequency of the bilayer is lower than reported in the literature with Raman spectroscopy.^17^ This discrepancy can be attributed to a different substrate, which influences the strain in the WS_2_, or to different temperatures. Upon laser excitation, the temperature of the WS_2_ layer increases, which reduces the phonon frequency. This temperature-dependent frequency shift has been shown for higher frequency phonons, but the reported shifts are relatively small.^34^ The effect of the substrate on which the nanolayered WS_2_ crystals can be deposited is considered by applying a biaxial strain in the basal *xy* plane through rescaling the relaxed cell vectors. Both modes increase in energy when under compressive strain (Figure 5) demonstrating that strain-induced effects from a substrate can effectively change the breathing mode energy. The soft transfer technique used, in combination with leaving the WS_2_ crystal on the PDMS substrate, has most likely resulted in a very small strain on the crystal and therefore a lower phonon frequency with respect to the values reported earlier.^10,17^

The origin of the change in the photoinduced sheet conductance at the phonon frequency is unclear. A possible explanation could be the interaction between layers at the phonon frequency, similarly to the increase observed in magnetic perovskite structures.^35^ In order to fully describe this behavior, electron–phonon matrix element calculations would be needed.

## Conclusions

Using microstructured THz photoconductive antennas and photoexcitation of an exfoliated nanolayered WS_2_ crystal, we have retrieved the THz sheet conductance of a bilayer and two thicker layers of 14 and 30 nm. The measurements show a Drude–Smith conductance and coupling of the carriers to a phonon mode. DFT calculations reveal that this phonon mode corresponds to the breathing mode in the van der Waals bound semiconductor, showing a strong dependence on the strain. Time-resolved THz spectroscopy represents a viable technique for mapping transient charge transport in photoexcited samples, enabling the non-invasive investigation of nanoscale materials relevant for two-dimensional nanodevices.

## Methods

### Time-Resolved THz Spectroscopy

A Coherent Astrella amplified laser system is used for performing time-resolved THz near-field spectroscopy. The laser generates *λ* = 800 nm pulses with a duration of 100 fs and an intensity of 1.2 mJ per pulse at a repetition rate of 5 kHz. The pulse is split into three beams, for THz generation, THz detection, and optical excitation. An intensity of 0.5 mJ per pulse is used for generating the THz radiation in a ZnTe crystal by optical rectification that is sent through the sample.^13^ The transmitted THz radiation is detected at a distance of 1 *μ*m from the surface of the sample with a commercially available microstructured photoconductive antenna (TeraSpike TD-800-X-WT, Protemics GmbH). The THz pulse is measured in the time domain by delaying the optical probe beam with respect to the THz pulse. The detector is only active for a considerably shorter time than the length of the THz pulse. Therefore, the full THz transient is retrieved by scanning the probe delay stage and measuring a small portion of the THz pulse at each position. The sample is excited by a frequency-doubled pulse of λ = 400 nm with an optical fluence of 20 μJ/cm^2^. The arrival time of the probe beam for THz detection and the pump beam for optical excitation can be varied with respect to the THz pulse with the probe and pump delay stages, respectively. To ensure that every measured portion of the THz pulse encounters the same pump delay time, the pump delay stage is moved in identical steps as the probe delay stage. A dual modulation scheme with lock-in detection is used to simultaneously retrieve the THz transmission, as well as the influence of the photoexcitation on the THz transmission.^36^

### Ab Initio DFT Calculations

Phonon energy calculations were performed using the finite displacement method within the harmonic approximation as implemented in the Phonopy package.^37^ For our calculations, we adopted projector augmented wave-Perdew–Burke–Ernzerhof exchange–correlation functionals with a 8 × 8 × 1 *k*-point mesh in a reciprocal space generated according to the Monkhorst–Pack scheme. A conventional unit cell for the 2H-polytype bilayer with two W and four S atoms (Figure 4a) was used in all calculations with a plane wave energy cutoff of 450 eV and an energy convergence criteria of 10^–6^ eV. Furthermore, we employed a 4 × 4 × 1 supercell of the conventional unit cell with a vacuum spacing of 14 nm along the *z*-axis to perform lattice relaxation and finite displacements.

## Supporting Information Available

Photoluminescence measurements and atomic force microscopy measurements (PDF)
