# Supplementary material for: High-Frequency Sheet Conductance of Nanolayered WS2 Crystals for Two-Dimensional Nanodevices
Source: ACS Appl Nano Mater. 2022 Oct 13;5(10):15557–62. doi: 10.1021/acsanm.2c03517 (PMC9623546; doi:10.1021/acsanm.2c03517)
Supplement: Supplementary file 1 — an2c03517_si_001.pdf [file an2c03517_si_001.pdf]

# Supporting information

## High-Frequency Sheet Conductance of Nanolayered $WS_2$ Crystals for Two-Dimensional Nanodevices

Stan E.T. ter Huurne,[*,†] Adonai Rodrigues Da Cruz,[*,†] Niels van Hoof,[†] Rasmus H. Godiksen,[†] Sara A. Elrafei,[†] Alberto G. Curto,[†] Michael E. Flatté,[‡,†] and Jaime Gómez Rivas[*,†]

†*Department of Applied Physics and Eindhoven Hendrik Casimir Institute, Eindhoven University of Technology, P.O. Box 513, 5600 MB Eindhoven, The Netherlands.*

‡*Department of Physics and Astronomy, University of Iowa, Iowa City, IA 52242, USA*

E-mail: s.e.t.t.huurne@tue.nl; a.rodrigues.da.cruz@tue.nl; j.gomez.rivas@tue.nl

# Contents

**SI.1 Photoluminescence measurements** **S2**

**SI.2 Atomic force microscopy measurements** **S3**

## SI.1 Photoluminescence measurements

The photoluminescence (PL) measurements were acquired with a microscope in epi-fluorescence geometry. We excited the bilayer with a 532 nm continuous wave laser through a 40x, 0.6 NA objective with a 8 $\mu$W power before the objective lens. The PL signal is filtered from the laser and goes through a 50 $\mu$m fiber to an Andor Shamrock 303 spectrometer with an Andor Newton EMCCD camera to obtain the PL spectrum. The spectra of the substrate and the thin $WS_2$ layer are shown in Fig. SI.1, the indirect peak at 705 nm matches with the emission from a bilayer.

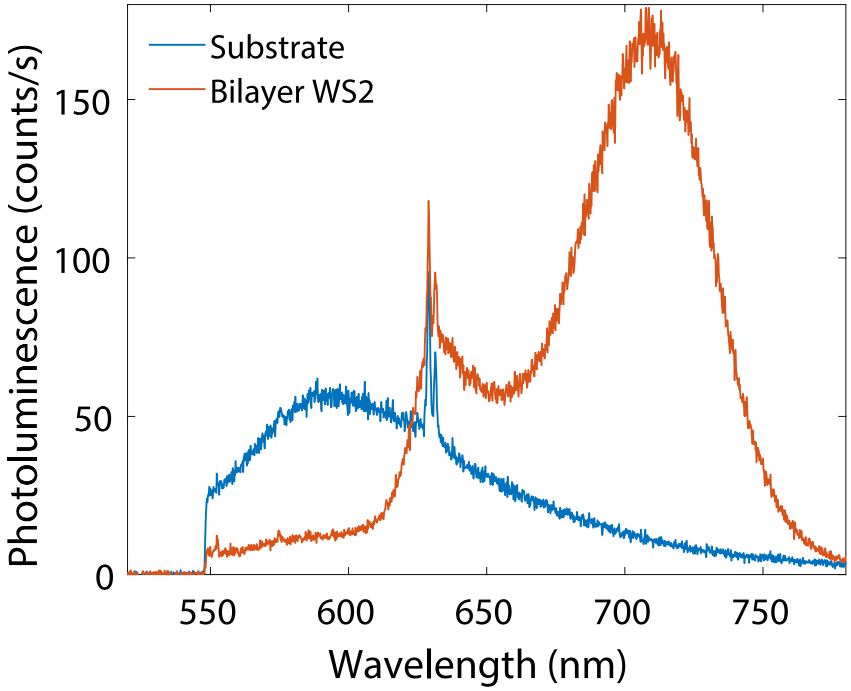


Figure SI.1: Photoluminescence spectra obtained from the substrate (blue) and the $WS_2$ bilayer (red). From the wavelength and linewidth of the exciton at 630 nm and the indirect peak at 705 nm the thickness can be determined to be a bilayer.

## SI.2 Atomic force microscopy measurements

The sample was characterized using an atomic force microscope (AFM), Scan Asys Dimension Icon Bruker, to measure the change in relative height (the layers thickness) over the sample. The atomic force microscopy measurements were performed in two steps and are shown in Fig. SI.2. The first step is from the bilayer region to the adjacent layer (in blue). The average step size is 12.7 nm, so a thickness of 14 nm is determined. The second step is from the 14 nm region to the thickest region that has been measured (in red). The average step size is 16 nm, so a thickness of 30 nm is determined.

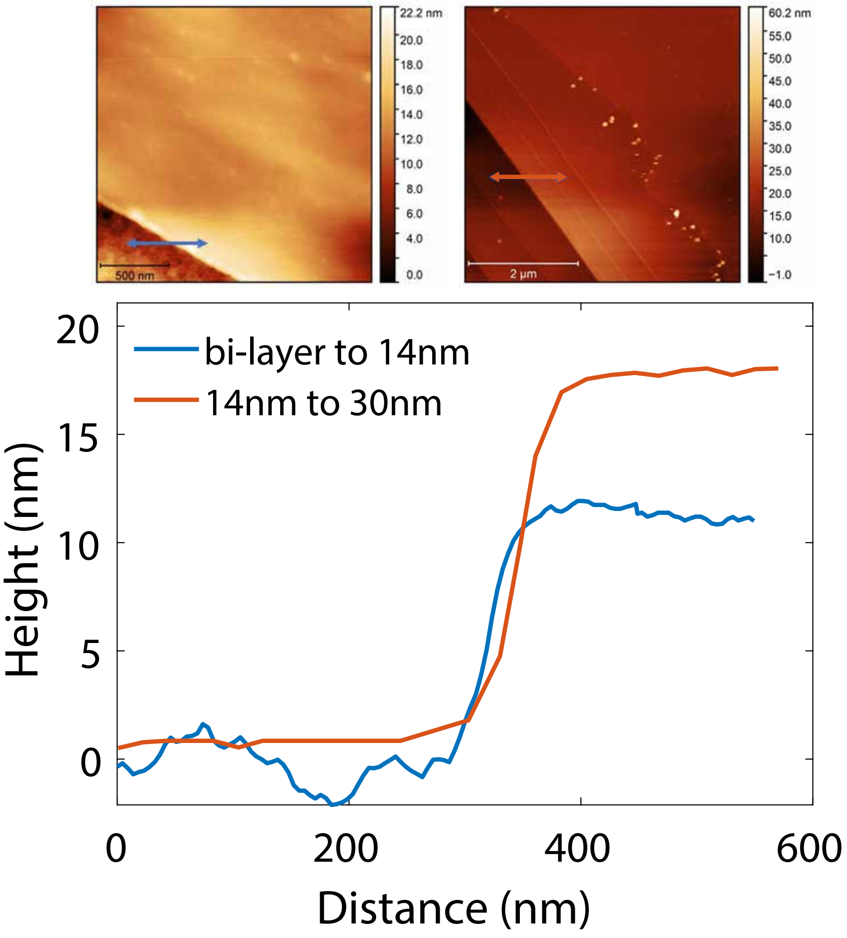


Figure SI.2: Atomic force microscopy measurements of the interface between the bilayer and 14 nm thick region and between the 14 nm to 30 nm thick region and a linescan of the two interfaces.
